# Genome Assemblies of Three Soil-Associated *Devosia* species: *D. insulae*, *D. limi*, and *D. soli*

**DOI:** 10.1128/genomeA.00514-15

**Published:** 2015-05-21

**Authors:** Yousef I. Hassan, Dion Lepp, Ting Zhou

**Affiliations:** Guelph Food Research Centre, Agriculture and Agri-Food Canada, Guelph, Ontario, Canada

## Abstract

Agricultural soils constitute highly diverse ecosystems with very rich bacterial populations. Recent studies employing next-generation sequencing techniques have begun to explore the dynamics of bacterial species of such soils and utilized metagenomics approaches to understand how the diversity in soil microorganisms is affected or modified by agricultural practices. Understanding any microorganism’s environmental adaptability in the genomic era starts by fully appreciating their encoding genome. Here, we report the draft genome sequences of three *Devosia* species based on three type strains that originated from soil samples: *D. insulae* strain DS-56, *D. limi* strain DSM17137, and *D. soli* strain GH2-10.

## GENOME ANNOUNCEMENT

Agricultural soils constitute highly diverse ecosystems with rich bacterial populations (1, 2). Recent studies have begun to explore the dynamics of bacterial species of such soils and utilized metagenomics approaches to understand how the diversity in soil microorganisms is affected by agricultural practices (3).

The first deposited *Devosia* species can be tracked to the 1940s, when it was characterized as *Pseudomonas riboflavina* (4). The reclassification of that isolate added the designation *Devosia* to a growing list of microorganisms emerging form soil samples (4). Since then, more than 16 different *Devosia* species have been validated (5–18). The majority of the reported isolates affiliated with this genus originate from soil samples, such as native forest and agricultural soils (19–21). Several studies have also reported on the ability of *Devosia* spp. to adapt to challenging ecological niches, including soils contaminated with diesels, hydrocarbon pesticides, and mycotoxins-enriched soils (9, 13, 22).

Understanding any microorganism’s environmental adaptability starts by appreciating their encoding genome. For this reason we are reporting the draft genome sequences of three *Devosia* species that originated from soil samples, based on three type strains: *D. insulae* strain DS-56 isolated from Dokdo Island, East Sea of Korea (17); *D. limi* strain DSM17137 from a nitrifying inoculum (14); and *D. soli* strain GH2-10 from a greenhouse soil used for cultivating lettuce in Daejeon City, Korea (16).

The type strains *D. insulae* strain DS-56 (DSM17955), *D. limi* strain DSM17137 (DSM17137), and *D. soli* strain GH2-10 (DSM17780) were obtained from DSMZ (Braunschweig, Germany) and were reactivated according to the supplier’s recommendations. The genomic DNA was prepared using Puregene yeast/bacteria kit B (Qiagen, Canada). Sequencing libraries were generated using the Nextera XT kit (Illumina, San Diego, USA), and 300-bp paired-end sequencing was performed with a MiSeq benchtop sequencer using a 600-cycle version 3 kit (Illumina). Following demultiplexing and quality filtering by MiSeq control software version 2.5.0.5, human- and phiX-contaminated reads were removed by mapping the filtered reads against the respective genomes using BBMap version 34.65 (http://sourceforge.net/projects/bbmap/). The average lengths of the final reads were *D. limi* (292 bp), *D. soli* (289 bp ), and *D. insulae* (284 bp). The sequencing depths were 279×, 185×, and 70× for *D. insulae*, *D. limi*, and *D. soli*, respectively.

Reads were assessed for quality with FastQC version 0.10.1 (Babraham Bioinformatics, United Kingdom) and assembled into contigs with the SPAdes assembler version 3.0.0 (23). Assemblies qualities were assessed with Quast version 2.3 (24).

The above efforts yielded three different draft genomes with varying sizes: 3,841,237 bp for *D. insulae*, 4,136,371 bp for *D. soli*, and 4,474,336 bp for *D. limi*. The *N*_50_ for the deposited assemblies were 131,891 bp (*D. limi*), 378,020 bp (*D. soli*), and 314,915 bp (*D. insulae*), while the total numbers of contigs were 158 for *D. limi*, 48 for *D. soli*, and 131 for *D. insulae*, respectively.

### Nucleotide sequence accession numbers.

The above-reported genomes were deposited in DDBJ/EMBL/GenBank under the following accession numbers: LAJE00000000 (*D. insulae* strain DS-56), LAJF00000000 (*D. limi* strain DSM17137), and LAJG00000000 (*D. soli* strain GH2-10).
